# Lessons from the 2024–2025 measles outbreak in southern Viet Nam

**DOI:** 10.5365/wpsar.2026.17.2.1326

**Published:** 2026-04-20

**Authors:** Thang Nguyen-Tien, Hoa Minh Tran, Anh Vu Le

**Affiliations:** a Vietnam Public Health Association, Ha Noi, Viet Nam.; b University of Texas Medical Branch, Galveston, Texas, United States of America.; c Hong Bang International University, Ho Chi Minh City, Viet Nam.; d Dong Nai Center for Diseases Control, Dong Nai, Viet Nam.; e Dong Nai Provincial Public Health Association, Dong Nai, Viet Nam.

Control of measles outbreaks can be ensured only when herd immunity is greater than 95%. (*1*) There is no specific treatment, but vaccination is the most effective public health intervention that has saved millions of lives. (*2*) Recently, many countries worldwide have experienced a resurgence of measles. (*3*-*5*)

In 2024, Viet Nam experienced an increase in measles cases, with the southern province of Dong Nai reporting one of the worst outbreaks. Dong Nai covers 5897.8 km^2^, with approximately 3.1 million residents, including approximately 246 000 children aged < 5 years. (*6*) The province has 2258 health-care facilities and 17 503 health workers. Its numerous industrial zones and high migrant population pose challenges for childhood vaccination campaigns.

In Viet Nam, measles cases are classified for surveillance as suspected (clinical) or confirmed. A suspected case is defined as a patient with a history of contact with a confirmed case or residence in an endemic area, who presents with fever and rash, and one of the following symptoms: cough, runny nose, conjunctivitis, Koplik spots, lymphadenopathy, joint swelling or pain. Confirmed cases are identified through laboratory testing, including enzyme-linked immunosorbent assay detecting measles-specific immunoglobulin M antibodies or polymerase chain reaction. Under routine surveillance procedures, suspected cases are reported to the health information system and investigated by the Dong Nai Provincial Center for Disease Control (CDC), with specimens sent to the Pasteur Institute in Ho Chi Minh City for confirmation.

Surveillance data from Dong Nai CDC indicated that first-dose measles vaccination coverage was 73.1% in 2021 and 73.9% in 2022. Although coverage increased to 91.9% in 2023, it remained below the recommended herd immunity threshold. Dong Nai observed sporadic measles cases initially in mid-2024, accelerating during the fourth quarter of the year. Reported cases peaked in week 50 and 51 with around 900 cases per week, totalling 7173 cases in 2024. Cases were high during the first 2 weeks of 2025, at more than 800, and then began to decline, from approximately 600 cases in week 3 to 80 cases in week 14. Among the recorded cases, 55.9% (6388/11 434) occurred in children aged < 5 years, 24.3% (2778/11 434) in those aged 5–10 years, and 19.8% (2268/11 434) in individuals aged > 10 years.

In accordance with the World Health Organization's *Measles outbreak guide*, (*7*) Dong Nai CDC implemented context-appropriate response strategies. The first strategy was to determine the most high-risk age groups and then prioritize immunization in children aged 0–10 years. The second strategy was to promote catch-up immunization by actively engaging with communities and offering vaccinations at several locations, including commune health stations, district health centres, hospitals and schools, and by using community health volunteers to identify unvaccinated children.

Additional successful measures taken during the preparedness phase included strong leadership, efficient policy coordination among the national and provincial governments, and utilization of mobile vaccination teams trained annually to vaccinate inaccessible populations.

These measures helped bring the outbreak under control. Immunization campaigns conducted from 27 September 2024 to 24 March 2025 achieved 97.5% coverage (112 340/115 209 people at risk), including health staff (4369/4408) and children aged 0–10 years (107 971/110 801), which was above the level required for herd immunity.

To describe the epidemiology of the measles outbreak and forecast trends in Dong Nai for the remainder of 2025, we fitted several autoregressive integrated moving average (ARIMA) models and selected an ARIMA (0,1,1) model based on the best fit to weekly case data (**Fig. 1**). The model captured outbreak progression and projected a continued decline or stabilization in cases. However, widening forecast confidence intervals indicated increasing uncertainty over time, underscoring the need for continued surveillance in longer term public health planning.

**Fig. 1:**
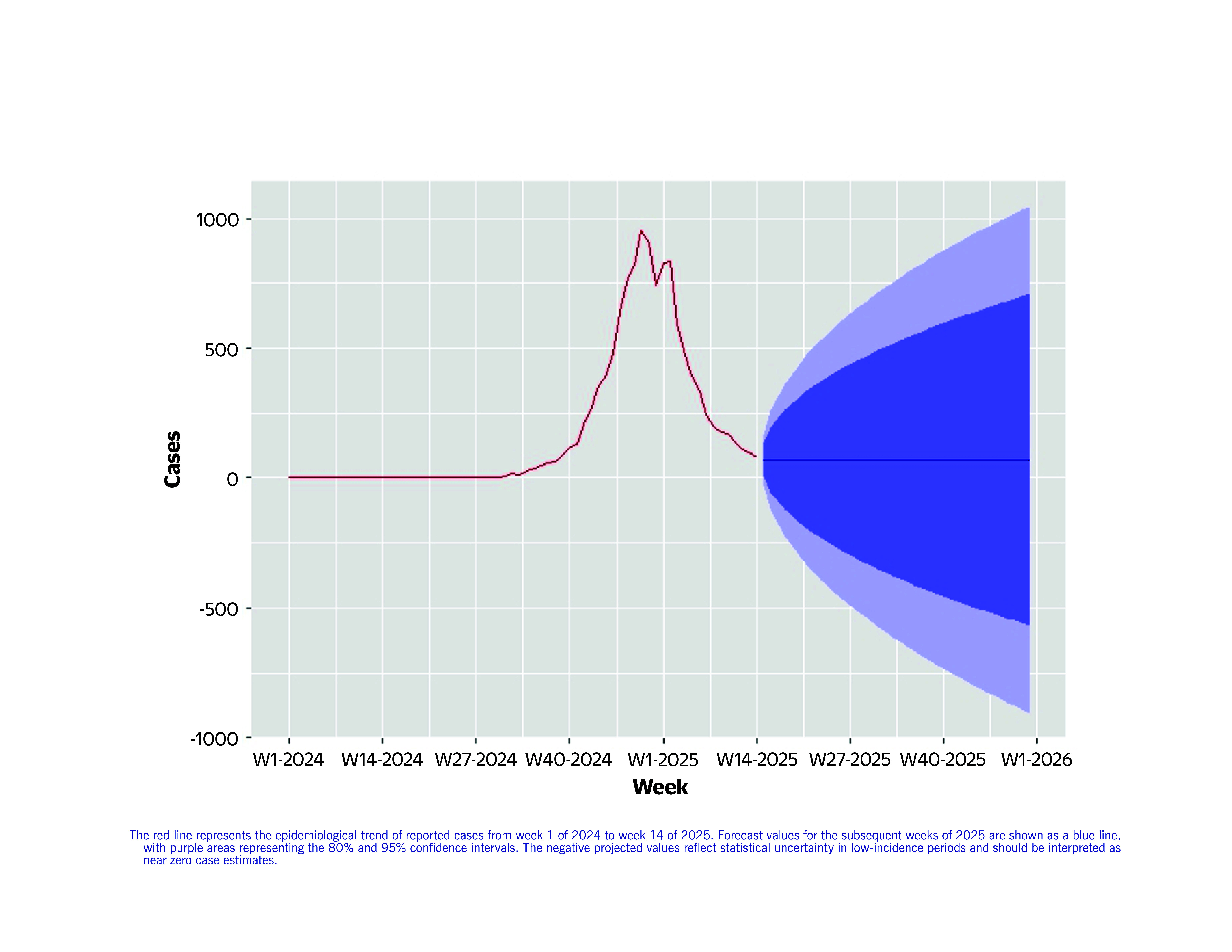
Observed and projected trends in measles cases in Dong Nai province from week 1 of 2024 to week 52 of 2025 using the ARIMA (0,1,1)

[**insert** Figure 1]

Despite these achievements, several root causes of the outbreak were recognized once a summary after-action report was prepared by Dong Nai CDC:

Failure to vaccinate: The COVID-19 pandemic disrupted routine immunization services, particularly among migrant populations. (*8*, *9*)
High mobility and tracking challenges: Dong Nai's large industrial zones have a high migrant population, facilitating rapid outbreak spread and complicating immunization tracking. (*10*) Many parents did not have vaccination records, and children born before 2017 were not registered in the National Immunization Information Management System, delaying identification of targets for vaccination.
Delayed surveillance and operation: Initial cases in weeks 33–39 of 2024 were not promptly investigated, delaying outbreak declaration and catch-up immunization activities.
Policy limitations: Unclear policies between the health and finance ministries related to health-worker per diem hindered outbreak planning and resource mobilization.
Obstacles to resources and communication: The campaigns relied on residential health volunteers to collect information about targeted groups, especially in large communes, where this proved time-consuming. Communication between commune health stations, schools and families was inconsistent. Instructions were often sent informally via social messaging apps, which led to missed or inaccurate information.
Vaccination misinformation and hesitancy: Social media misinformation significantly reduced vaccine coverage and increased hesitancy. (*11*)

## Take-home messages and recommendations

Enhancing routine immunization is crucial in preventing future measles outbreaks. Recommendations from this analysis include:

Accelerate catch-up campaigns through school-based delivery, mobile clinics and child health weeks, especially in low-coverage areas.
Foster multisectoral coordination, particularly with the education and health sectors, to identify undervaccinated children. Active engagement with primary schools and kindergartens is key to improving outreach.
Boost digital communication interventions and public awareness campaigns. Disseminate clear, concise, accurate messages about vaccine safety via social media, SMS and community influencers. Use targeted information campaigns in schools to promote vaccine acceptance and address misinformation.
Strengthen surveillance and immunization information systems, especially for high-risk and migrant populations.
Standardize risk communication protocols. Develop standardized communication protocols for health centres, schools and families to reduce information loss and improve coordination.

Our focus was on how health-system resilience and community engagement interact in measles outbreak control. Overcoming systemic and behavioural barriers, such as surveillance gaps, strengthening cross-sectoral partnerships and leveraging community trust are central to preventing future outbreaks and progressing towards measles elimination in Viet Nam and the Western Pacific Region.
